# Influence of Electrolyte Additives on Interfacial Stability of Manganese-Rich Lithium-Ion Battery Cathodes

**DOI:** 10.1021/acsaem.5c00862

**Published:** 2025-08-06

**Authors:** Nikita S. Dutta, Madison King, Bingning Wang, Chen Liao, John S. Mangum, Donal P. Finegan, Bertrand J. Tremolet de Villers, Katherine Jungjohann

**Affiliations:** † Materials, Chemistry, and Computational Sciences, 53405 National Renewable Energy Laboratory, Golden, Colorado 80401, United States; ‡ Center for Materials Interfaces in Research and Applications, Northern Arizona University, Flagstaff, Arizona 86011, United States; § Chemical Sciences and Engineering Division, 1291 Argonne National Laboratory, Lemont, Illinois 60439, United States

**Keywords:** lithium-ion battery, earth-abundant cathode, cathode electrolyte interphase, cryo-electron microscopy, electron energy loss spectroscopy

## Abstract

Affordable, long-lasting energy storage has become critical to support increased electricity demand in recent years. Cobalt-free, lithium- and manganese-rich lithium nickel manganese oxide (LMR-NM) cathodes stand to reduce cost and supply-chain concerns associated with traditional cobalt-containing cathodes for lithium-ion batteries by leveraging more earth-abundant materials; however, they have shown issues with long-term cycling stability. Here, we investigate lithium difluoro­(oxalate)­borate (LiDFOB), tris­(trimethylsilyl) phosphite (TMSPi), and vinylene carbonate (VC) electrolyte additives for their ability to improve cycling performance of LMR-NM (0.3 Li_2_MnO_3_ + 0.7 LiMn_0.5_Ni_0.5_0_2_) cells. Cryogenic scanning transmission electron microscopy (cryo-STEM) with electron energy loss spectroscopy enables the construction of a structure–function relationship between cathode electrolyte interphase (CEI) characteristics and the electrochemical performance of cells aged with these additives. We find the combination of 2 wt % TMSPi + 1 wt % LiDFOB performs better than any single additive, achieving a 28% improvement in specific capacity over the baseline electrolyte after long-term cycling. We attribute this to LiDFOB mitigating Mn ion dissolution, with cryo-STEM showing Mn stabilized up to the CEI surface, coupled with improved CEI structure and chemistry enabled by TMSPi, evidenced by a moderately thick (∼7–15 nm) CEI that appears to protect against further electrolyte reactions with the particle. These results, achieved through site-specific nanoscale characterization, directly reveal mechanisms through which electrolyte engineering can improve the performance of earth-abundant cathodes, enabling informed development of more affordable and reliable batteries to meet future energy storage needs.

## Graphical abstract

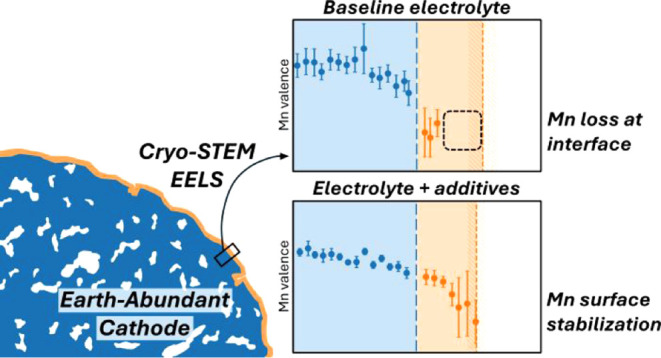

## 1 Introduction

Increased electricity demand and distributed generation have led to a greater need for accessible and reliable rechargeable energy storage solutions. State-of-the-art lithium-ion batteries (LIBs) rely on cobalt-containing cathodes,^1^ but cobalt is an expensive, toxic, and limited resource.^2,3^ Cobalt-free, lithium- and manganese-rich lithium nickel manganese oxide (LMR-NM) cathodes offer an alternative solution utilizing more earth-abundant, lower-cost materials.^3,4^ However, while these earth-abundant cathode alternatives provide high energy densities, they show issues with high voltage fade and reduced long-term cycling stability compared to their cobalt-containing counterparts.^4,5^ This performance degradation is partly caused by electrolyte decomposition, inducing delamination, pitting, and transition metal ion dissolution.^6–8^

Previous studies have shown that these types of detrimental reactions can be reduced or eliminated in cobalt-containing systems through changes in electrolyte composition.^9^ Lithium difluoro­(oxalate)­borate (LiDFOB), tris­(trimethylsilyl) phosphite (TMSPi), and vinylene carbonate (VC) are common additives that have shown increased performance in lithium nickel manganese cobalt oxide (NMC) cells,^10–16^ including LMR-NMC.^17^ LiDFOB forms an inorganic-rich cathode electrolyte interphase (CEI) that improves cycling stability and rate capabilities;^10,11,18^ TMSPi scavenges hydrofluoric acid (HF), helping to reduce transition metal ion dissolution and increase cycling stability;^12–14,19,20^ and VC has significant impacts on anode stabilization through solid electrolyte interphase (SEI) formation.^15,16^ Much less work has been done to explore the influence of these additives on cobalt-free systems, but they have shown promise; Wang et al. found LiDFOB reduces Mn ion dissolution in LMR-NM but argued that this was due to suppression of electrolyte degradation rather than formation of a passivating CEI,^21^ and the combination of LiDFOB and TMSPi was found to enhance the electrochemical performance of cobalt-free spinel cathodes as well.^6,22^

Determining the precise mechanisms underlying these performance enhancements requires a detailed understanding of the cathode surface chemistry and CEI, which are heterogeneous at the nanoscale.^23^ Prior work has typically studied the effects of these additives with techniques such as X-ray photoelectron spectroscopy (XPS)^10–12,14,17^ or nuclear magnetic resonance spectroscopy^6,13,15,21^ which offer excellent chemical resolution but are limited in spatial resolution. Thus, while these tools provide valuable information about species present at the surface of the cathode, our understanding of local chemistry within the CEI or at the surface of individual cathode particles remains incomplete.

Nanoscale characterization of cycled battery materials has historically been challenging due to the electron beam-sensitive nature of the CEI and SEI, which complicates the use of traditional scanning transmission electron microscopy (STEM) techniques. However, cryogenic STEM (cryo-STEM) has become an increasingly popular tool to study electrode cross-sections prepared by cryogenic focused ion beam (cryo-FIB) without losing crucial information at delicate interfaces to beam damage.^24–26^ Here, we apply cryo-STEM to study local chemistry at the surface of cathode particles cycled with a range of electrolyte additives. Electron energy loss spectroscopy (EELS) can then be coupled with cryo-STEM to gain detailed information on the interfacial chemistry of cycled cathodes, considering individual particle surfaces at the nanoscale.^27,28^ Regarding cycled electrode surfaces, previous work from Zhang et al. represents one of the first set of studies of this kind on surface characterization from cycled anode nanoparticles^28^ and cathode nanoparticles.^29^ No published papers have analyzed cathode electrode surfaces extracted from working coin cells using cryo-FIB and cryo-STEM/EELS.

We use these techniques to study LMR-NM cathodes cycled with various electrolyte additives (VC, LiDFOB, TMSPi, and TMSPi + LiDFOB) to explore the potential for performance enhancement in earth-abundant LIBs via electrolyte engineering. The electrochemistry of the LMR-NM full cells is measured through a well-established protocol,^30^ and the 93rd cycle performance and subsequent impedance measurement are used as criteria for comparing the performance between these electrolyte additives. VC is observed to provide only a minor increase in specific capacity and a decrease in impedance over the baseline electrolyte after 93 aging cycles, while LiDFOB and TMSPi each offer substantial improvements. Performance is further enhanced by combining these two beneficial additives to achieve a 28% increase in specific capacity over that of the baseline electrolyte. Cryo-STEM with EELS is then used to explore the mechanisms underlying these effects. We find that LiDFOB mitigates Mn dissolution from the CEI, while TMSPi contributes to optimal CEI thickness and chemistry. In combination, these additives produce a CEI of moderate thickness, with Mn stabilized up to the CEI surface, that appears to have passivated further electrolyte reactions with the particle. Thus, these results demonstrate that this combination of electrolyte additives improves the electrochemical performance of earth-abundant cathodes via enhanced interfacial stability at the nanoscale.

## 2 Results and Discussion

Full cells of LMR-NM (0.3 Li_2_MnO_3_ + 0.7 LiMn_0.5_Ni_0.5_0_2_) versus graphite (Gr) were prepared using five different electrolyte formulations. The Gen2 electrolyte [1.2 M LiPF_6_ in ethylene carbonate (EC):ethyl methyl carbonate (EMC) (w/w 3:7)] was used as a baseline and then coupled to Gen2 + 1 wt % VC, Gen2 + 2 wt % TMSPi, Gen2 + 1 wt % LiDFOB, and Gen2 + 2 wt % TMSPi + 1 wt % LiDFOB. As indicated in Section 4.1, an extra-thick graphite anode was used to maintain a high ratio between the negative electrode energy capacity and the positive electrode energy density (N/P ratio), even in the cycles of activation, where specific capacities tend to surpass the specific capacities observed during subsequent aging cycles. Particularly, area-specific capacities of the anode and cathode are reaching 3.16 mAh/cm^2^ with a loading of 9.9 mg/cm^2^, much larger than the previous standard of 1.93 mAh/cm^2^ with a loading of 6.36 mg/cm^2^. The conditions regarding the anode, including processing and electrochemical cycling to enable efficient SEI formation, are similar to those in previous reports. Figure 1a shows charge and discharge profiles for the first cycle and 93rd aging cycle for all samples; the full cycling protocol is given in Section 4. The cycling protocol was designed to provide not only the electrochemical properties with respect to specific capacity but also a holistic view of both the SEI, CEI, and impedance increase through postmortem characterization. The uniqueness of the activation cycle for LMR-NM requires formation cycles not only for electrolyte additives but also for these cobalt-free earth-abundant cathodes.^31^ The 93rd cycling performance comparison demonstrates capacity retention for each of the electrolyte additive cells.

**Figure 1:**
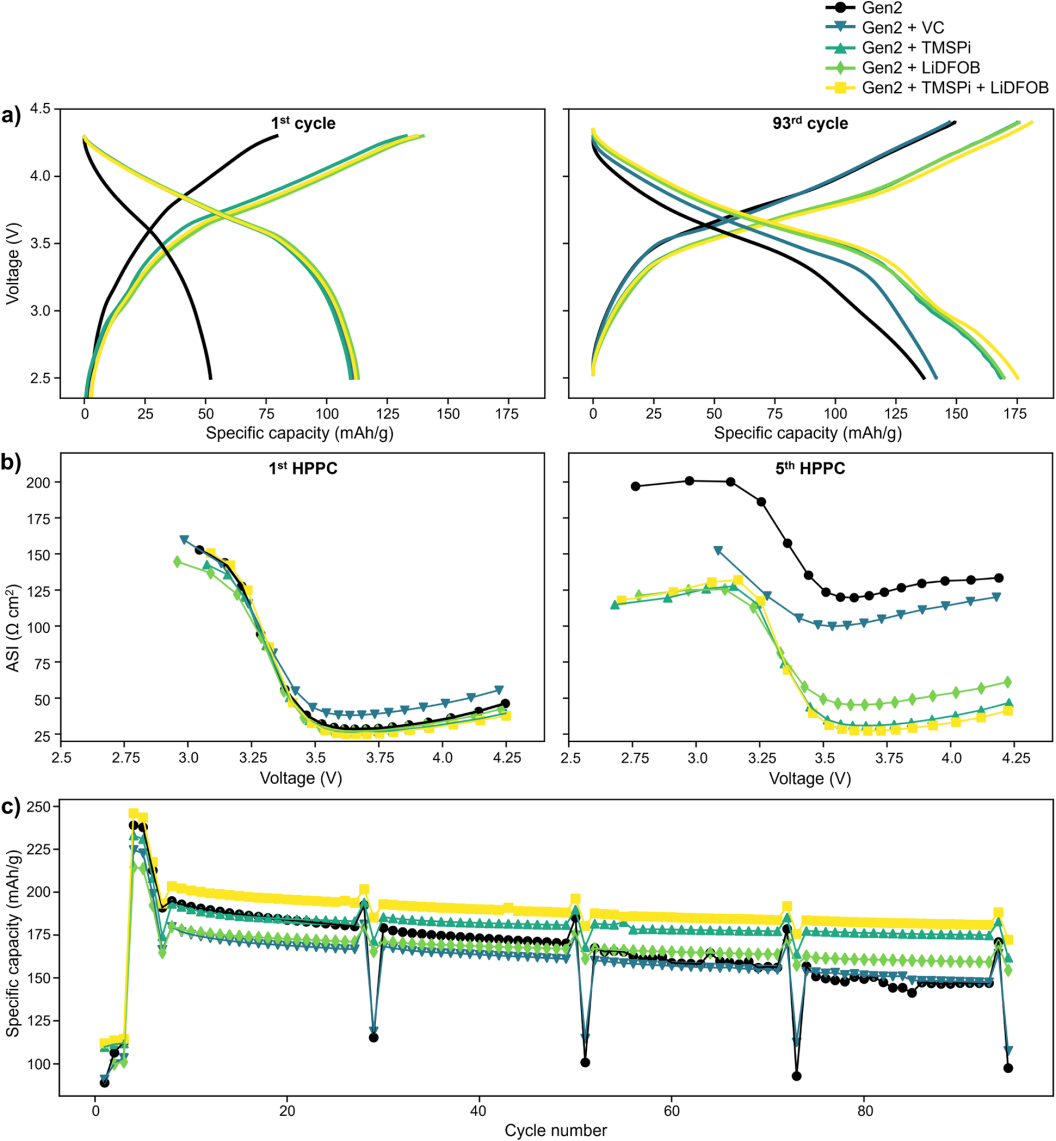
(a) First cycle (left) and 93rd cycle (right) charge and discharge profiles for Gr||LMR- NM cells cycled with the Gen2 electrolyte alone (black lines and circles), Gen2 + 1 wt % VC (blue lines and downward triangles), Gen2 + 2 wt % TMSPi (blue-green lines and upward triangles), Gen2 + 1 wt % LiDFOB (green lines and diamonds), or Gen2 + 2 wt % TMSPi + 1 wt % LiDFOB (yellow lines and squares). (b) Area-specific impedance for all samples measured by the first HPPC test (left; after cycle 6) and fifth HPPC test (right; after cycle 98). (c) Capacity retention for all samples over the full cycling protocol, as detailed in Section 4.

From both experimental^32^ and theoretical research,^33^ VC has been demonstrated to be an effective SEI component without much impact on the cathode. We sought to confirm this finding by adding 1 wt % VC to impact the formation of SEI on the graphite anode to reduce Mn transition metal deposition onto the graphite. After aging, the VC additive leads to only a minor increase in specific capacity over the Gen2 baseline, while TMSPi and LiDFOB both lead to significant increases of 23% and 24%, respectively. This improvement is further enhanced when using the latter two additives in combination, yielding a 28% improvement in specific capacity over the baseline electrolyte after aging, also apparent in the capacity retention plot in Figure 1c. Figure 1b shows area-specific impedance measured by the first and last hybrid pulse power characterization (HPPC) tests after formation cycling and aging, respectively. Here, the VC additive again shows a minor improvement over the Gen2 baseline after aging, while the TMSPi and LiDFOB additives each yield a significantly lower final impedance than the baseline electrolyte alone. However, while the charge and discharge profiles of Gen2 + 2 wt % TMSPi and Gen2 + 1 wt % LiDFOB are nearly indistinguishable after aging, the TMSPi additive leads to a notably lower final impedance. As with specific capacity, the two additives in combination show the greatest improvement in impedance over the Gen2 baseline after aging. These results demonstrate that these additives commonly explored for NMC can enhance the performance of LMR-NM cathodes as well.

To understand how surface and interfacial chemistry resulting from the electrolyte additives factor into these improvements in electrochemical performance, we employ cryo-STEM EELS on the cathodes removed and dried from coin cells after 100 cycles. The general method for EELS analysis is illustrated in Figure 2, with full details provided in Section 4. An EELS spectrum image is acquired at the surface of a particle within a thin cross-section of the cathode prepared by cryogenic focused ion beam (cryo-FIB) lift-out techniques.^34^ Low-temperature cross-sectional lamella preparation allows for the carbon, polyvinylidene difluoride (PVDF) binder, and delicate CEI structure to remain intact during ion beam milling and polishing. Overview cryo-STEM high-angle annular dark field (HAADF) images of cross-sections from all samples are given in Figure S1; each contains the surface of two LMR-NM cathode particles with a carbon PVDF binder in between. EELS maps were taken from at least three areas on the surface of each particle, with the goal of choosing areas that were sufficiently thin and, if possible, roughly evenly spaced along the particle surface. Segmentation is then applied to the HAADF signal in the EELS spectrum image to identify the particle surface, marked with a yellow line in Figure 2b, and pixels are binned by their distance from the surface, with positive distances defined as the particle interior and negative distances defined as the particle exterior, including the CEI. The red-shaded area in Figure 2b shows an example bin within the particle; Figure S2 further illustrates the image segmentation and distance map procedure. Finally, the EELS low-loss spectra from all pixels within a bin are summed to yield a higher signal-to-noise low-loss spectrum for each distance step from the particle surface. The EELS high-loss spectra from all pixels within a bin are summed in the same manner to produce a higher signal-to-noise high-loss spectrum for each distance step as well.

**Figure 2:**
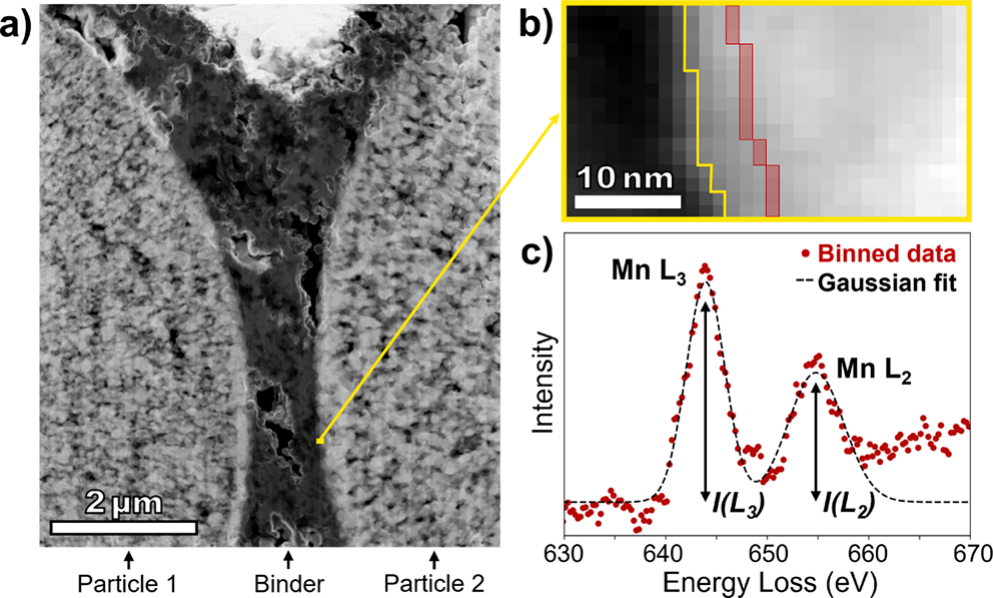
Illustration of the cryo-STEM EELS analysis process. (a) An EELS spectrum image is acquired at the surface of a cross-sectioned cathode particle. (b) The surface of the particle is identified by segmentation of the HAADF image (yellow line), and pixels are binned by distance from the surface (red shaded box). (c) EELS spectra from all pixels in a bin are summed. The background-corrected Mn L_2,3_-edge is fit to two Gaussians, and the ratio of their intensities is used to calculate the approximate Mn valence state.

We first analyze the Mn L_2,3_-edges in the binned data, which contain two peaks, or white lines, that arise from the excitation of core electrons to unoccupied d-orbitals and thus are sensitive to the Mn valence state. As illustrated in Figure 2c, the white lines can be fit by two Gaussian peaks, allowing for quantification of the energy difference and intensity ratio between the peaks, both of which are influenced by changes in the Mn oxidation state. Figure 3 shows the approximate Mn valence state versus distance from the particle surface for all samples, calculated using the Mn L_2,3_-edge intensity ratio and a calibration curve for mixed metallic Mn oxides from Loomer et al.^35^ The particle surface as determined by image segmentation is denoted by long dashed blue lines such that the blue data points within the blue-shaded areas correspond to the particle interior. In general, the Mn valence decreases from 15 nm within the particle to the surface.

**Figure 3:**
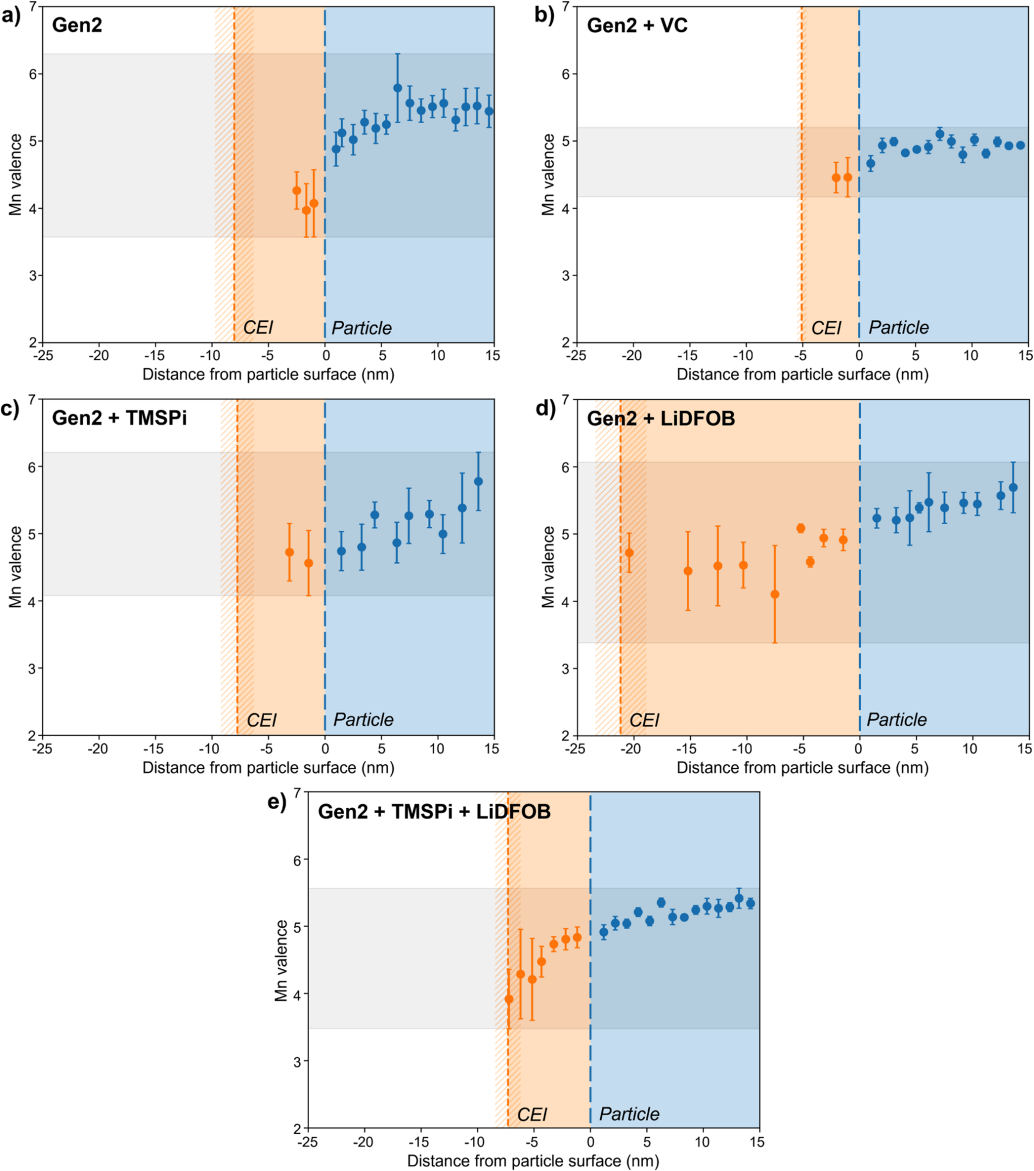
Approximate Mn valence states calculated using the Mn L_2,3_-edge intensity ratios for samples cycled in (a) Gen2 alone, (b) Gen2 + 1 wt % VC, (c) Gen2 + 2 wt % TMSPi, (d) Gen2 + 1 wt % LiDFOB, and (e) Gen2 + 2 wt % TMSPi + 1 wt % LiDFOB. Blue long dashed lines represent the particle surface as determined by segmentation of the HAADF images; blue circles within the blue shaded regions represent data from within the particle (defined as a positive distance from the surface). Orange short dashed lines represent the CEI surface, as determined by the average distance at which the O K-edge signal disappears; orange circles within the orange shaded regions represent data from within the CEI (defined as a negative distance from the surface). Each data point is the average of data from at least three different regions mapped with EELS, where error bars show the standard error (error bars in the x-direction are smaller than the marker). Hatched shaded orange regions around the orange dashed lines show the standard error of the average distance at which the O K-edge signal disappears. Gray shaded regions highlight the extent to which the calculated Mn valence varies over each sample. Note that the absolute value of the calculated valence depends on the reference chosen for calibration; more details on this effect can be found in the Supporting Information.

The CEI surface for each sample is estimated as the distance at which the K-edge of O disappears in the EELS data, since O is expected to be present within the CEI but not in the PVDF binder or carbon. This feature is denoted by short dashed orange lines, which show the average distance of the O K-edge signal drop-off over all spectrum images (representing 4–6 different regions of each sample). Orange data points within the CEI (the orange-shaded region) represent relative Mn valence values, which are generally lower relative to those within the particle, indicating that Mn at the surface of the LMR-NM particles is being reduced during cycling. In some cases, the Mn signal does not span the full thickness of the CEI, as evidenced by the absence of orange data points at the CEI/binder surface, suggesting that some reduced Mn has been lost to dissolution. This detailed analysis of Mn surface chemistry is supported by EELS maps showing the presence of other elements in the CEI, such as fluorine or boron; representative maps for each sample are given in Figure S3. Some transition metal segregation is observed in the maps for all samples; this is not clearly correlated with subsequent findings on the Mn surface chemistry but could be the subject of future study.

Overall, we observe that the sample cycled in the Gen2 baseline electrolyte alone (Figure 3a) shows significant variation of the Mn valence with the distance from the particle surface, highlighted with gray shading in Figure 3. There is also significant variation between regions of the sample, as evidenced by the error bars, which give the standard error of the mean of data from at least 3 different spectrum images, each capturing a different sample region. The sample shows evidence of Mn dissolution, as the Mn signal is not present in the outermost ∼5 nm of the estimated ∼8 nm thick CEI. In other words, the O and Mn EELS signals are not well colocated, with O present significantly further from the particle surface than Mn, suggesting Mn has been lost from the outermost surface of the CEI.

Of the four samples cycled with additives (Figure 3b–e), the samples containing LiDFOB are notable in that they do not show evidence of Mn loss from the CEI surface; rather, Mn is present to the same distance that O is present (Figure 3d,e). This suggests the LiDFOB additive is effective in reducing Mn dissolution, which has been supported by the previous literature attributing this effect to the suppression of acidic electrolyte decomposition products such as HF.^21^ However, the sample containing only LiDFOB as an additive shows large variation in the Mn valence with distance as well as between regions, and the CEI is significantly thicker than the other samples (∼21 nm). While the CEI thickness does vary when measuring samples taken from different parts of the cathodes (Figure S4), the CEI on the sample cycled in Gen2 + 1 wt % LiDFOB is the thickest observed for any sample. This offers an explanation as to why aging cycles in Gen2 + 1 wt % LiDFOB lead to higher impedance than cycling in Gen2 + 2 wt % TMSPi, despite similar increases in specific capacity over the baseline electrolyte (Figure 1b), as the thick CEI may lead to sluggish Li transport across the interface. In addition, EELS maps show the presence of boron not just in the CEI of this sample but also within the particle itself (Figure S3d), further suggesting that the CEI has failed to passivate reactions between the particle and electrolyte.

In contrast, the sample cycled in Gen2 + 2 wt % TMSPi shows a thinner CEI, along with less variation in the Mn valence with distance from the surface. In line with TMSPi’s reputation as an HF scavenger,^12–14^ EELS maps do not show significant fluorine in the CEI of this sample, although there does appear to be trace fluorine present within the particle (Figure S3c). This is in contrast to the EELS maps for all other samples, which do show fluorine components in the CEI, though this does not necessarily indicate HF presence. Importantly, unlike in the LiDFOB case, the sample still shows evidence of Mn loss from the outermost half of the CEI. Our previous work also indicates that LiDFOB alone suppresses electrolyte degradation, while aged TMSPi^36^ and LiDFOB^37^ together modify the CEI.

The sample cycled in Gen2 + 1 wt % VC (Figure 3b) shows Mn loss from roughly the outer half of its CEI as well. While the VC sample shows the least variation in Mn valence, both with a distance from the particle surface and between regions of the sample, possibly explaining the modest improvement in electrochemical performance observed over the Gen2 baseline, it also shows the thinnest CEI layer (∼5 nm) of all samples; it is possible that this thin CEI is insufficiently passivating, leading to poorer cycling behavior than the TMSPi sample, despite similar relative extents of Mn loss.

The complementary effects of the LiDFOB and TMSPi additives offer an explanation as to why the greatest improvement in specific capacity after aging over the baseline electrolyte is observed when using them in combination. The dual additive sample (Figure 3e) appears to combine the benefits of increased Mn retention due to LiDFOB, with a moderate CEI thickness and increased homogeneity in the Mn valence due to TMSPi. EELS maps also suggest an improved CEI chemistry with the combined additives; both fluorine and boron are present in the CEI but neither appear within the particle, suggesting the particle interior has been protected from further reactions with the electrolyte (Figure S3e). This combination of effects should lead to improved Li transport at both the cathode, due to the stable yet thin CEI that is formed, and at the anode. The retention of reduced Mn from the LMR-NM within the CEI also prevents the problematic deposition of Mn onto the anode, which was observed through inductively coupled plasma-mass spectrometry (ICP-MS) studies of the cycled graphite anodes (Figure S8). The Mn content in the electrolytes was not monitored, as Mn^2+^ (the soluble species) tends to be reduced on the graphite anode side.^38^ The observed increase in cathode stability is consistent with the significant decrease in cell impedance observed with the dual additives compared to the baseline electrolyte after aging and suggests that this overall increased cell performance is mainly affected by the CEI and not the SEI.

It should be noted that while the EELS analysis used here appropriately captures qualitative trends in the Mn valence within or between samples, using a literature calibration curve provides only an approximate value of the Mn valence, which is sensitive to the choice of reference and thus not quantitatively precise. Figure S5 shows analogous data to Figure 3, but with the Mn valence calculated using a range of calibration curves from the literature applied to either the Mn L_2,3_-edge intensity ratio or energy difference.^35,39,40^ As discussed in detail in the Supporting Information, the choice of reference influences the absolute value of the calculated valence but not general trends related to the extent of variation or Mn retention within samples. Likewise, when increasing the sampling area by preparing additional cryo-FIB cross sections from different parts of the cathodes (Figure S4), we still observe that the best-performing combination of additives enhances Mn retention at the surface of the CEI, compared to the Gen2 baseline where Mn loss is apparent, despite local variations in CEI thickness.

To probe further the differences in surface Mn retention between the various samples, we also consider the O K-edge structure. Previous studies have identified a pre-edge peak around 530 eV and related it to O-transition metal (TM) bonds.^41^ Figure 4 shows the background-corrected O K-edge (left plots) and Mn L_2,3_-edge (right plots), corresponding to increasing distance from the particle surface into the CEI, moving upward from the purple to yellow traces, for the Gen2 baseline sample and the best-performing combination of TMSPi and LiDFOB additives (analogous plots for the individual additives are given in Figure S6). For the sample cycled in the Gen2 baseline electrolyte alone (Figure 4a), the pre-edge peak is subtly apparent close to the particle surface but disappears by around 5 nm into the CEI, even though the step-like O K-edge is still apparent at farther distances. The Mn L_2,3_-edge appears shortly after this feature, around 6 nm from the surface, consistent with a lack of O-TM bonding. In contrast, the sample cycled in Gen2 + 2 wt % TMSPi + 1 wt % LiDFOB (Figure 4b) shows a pronounced pre-edge peak that persists until about 8 nm from the surface. This corresponds to a strong Mn L_2,3_-edge that disappears at the same distance as the pre-edge peak. Thus, the improved retention of Mn observed with the dual additives is correlated with the preservation of Mn–O bonds, suggesting this bonding may help to stabilize the valence of Mn at the CEI surface and prevent its dissolution.

**Figure 4:**
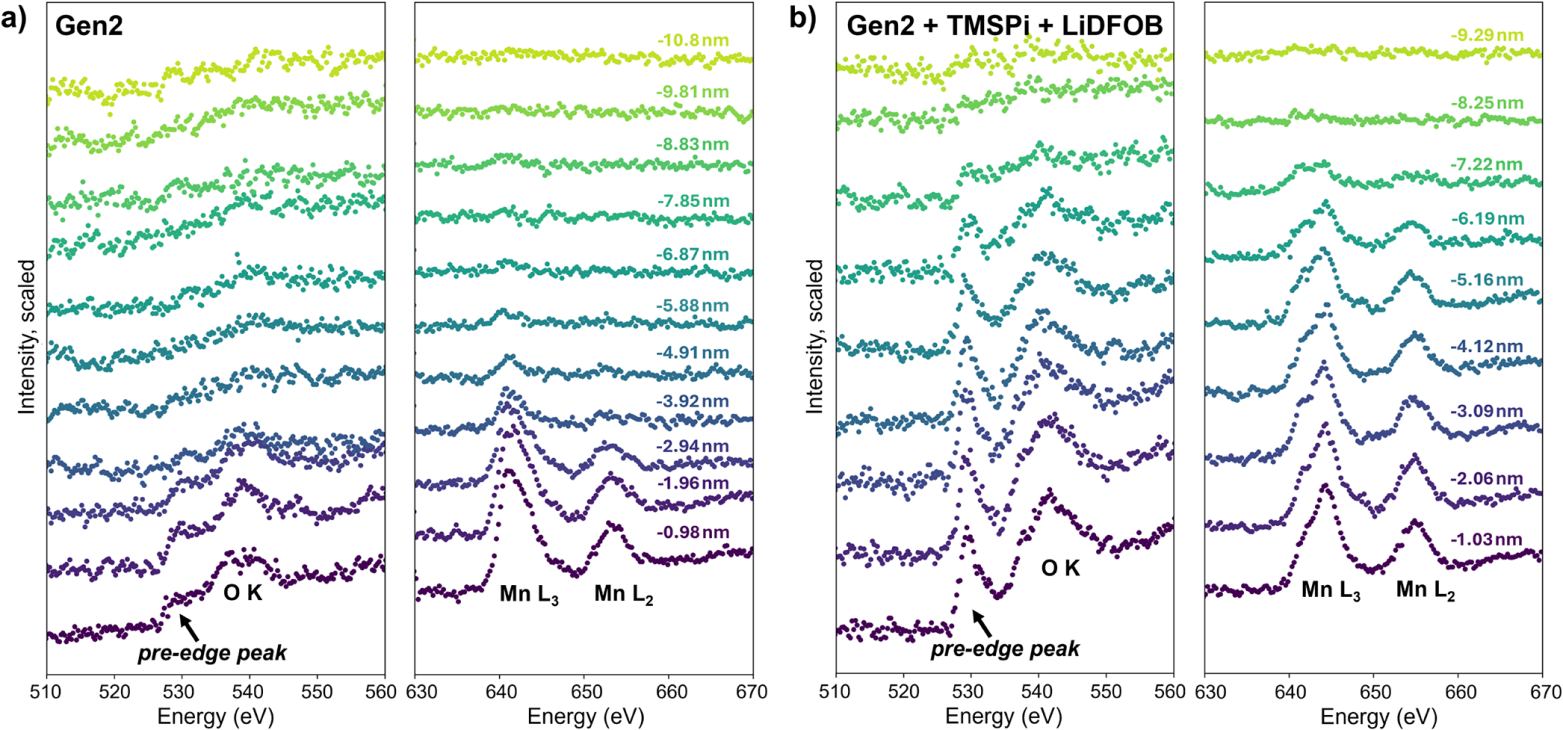
O K-edge (left) and Mn L_2,3_-edge (right) with increasing distance from the particle surface into the CEI for representative regions of samples cycled in (a) Gen2 alone or (b) Gen2 + 2 wt % TMSPi + 1 wt % LiDFOB. The pre-edge peak before the O K-edge (around 535 eV) is more prominent and present at further distances from the surface in the sample cycled with additives, correlated with a clear Mn L_2,3_-edge further from the surface as well, together suggesting that the additives enable improved Mn retention close to the CEI surface over the Gen2 baseline.

Together, these results demonstrate that the use of TMSPi and LiDFOB as electrolyte additives, either alone or in combination, improves the cycling performance of earth-abundant LMR-NM cathodes via enhanced interfacial chemical stability at the nanoscale. Our results suggest that LiDFOB plays a key role in mitigating Mn dissolution, despite forming a thick CEI that appears to be insufficiently passivating based on the presence of boron within the particle. This supports the suggestion put forward in the previous literature that the benefit of LiDFOB in reducing Mn dissolution from LMR-NM cathodes stems from its role in preventing electrolyte decomposition in the first place, rather than the ability to form a passivating CEI that protects against etching by decomposition products.^21^

The addition of TMSPi in combination with LiDFOB appears to preserve the benefits of Mn retention while also improving both the CEI thickness and chemistry, producing a boron-containing CEI that previous work has suggested plays a role in anchoring Mn to the surface of particles to prevent its dissolution.^42^ While VC does provide a moderate improvement in electrochemical performance, it does not appear to mitigate Mn dissolution or benefit the CEI and yields much smaller improvements in specific capacity and impedance post-aging over the Gen2 baseline than those achieved by LiDFOB and TMSPi. This suggests the latter additives’ role in stabilization of the cathode interface is more significant to enhancing the long-term cycling performance of the LMR-NM cells than VC’s potential impact on anode stabilization.

## 3 Conclusion

This work shows that TMSPi, LiDFOB, and, to a lesser degree, VC additives, which have been explored to improve cycling lifetimes of traditional NMC cathodes, also enhance the performance of Co-free LMR-NM cathodes through interfacial stabilization mechanisms revealed by site-specific, nanoscale cryo-STEM characterization. We find that LiDFOB is highly effective at mitigating Mn dissolution from the cathode, as evidenced by Mn stably bonded to O throughout the thickness of the CEI in cryo-STEM EELS maps; however, when used alone, it produces a thick (*>*20 nm), nonpassivating CEI that is correlated with higher impedance after aging than other additive formulations. TMSPi improves the CEI structure and chemistry, reducing the CEI thickness to contribute to lower impedance, but on its own fails to suppress Mn dissolution. We thus find that the best-performing combination of Gen2 + 2 wt % TMSPi + 1 wt % LiDFOB leads to a moderately thick (∼7–15 nm) CEI that retains Mn throughout its thickness and appears to protect against further electrolyte reactions with the particle. This improved interfacial structure and chemistry is correlated with decreased impedance and a 28% improvement in specific capacity after 93 aging cycles, compared to the baseline Gen2 electrolyte. These results, enabled through novel cryo-STEM techniques for interfacial characterization, will support the informed development of high-performing, earth-abundant, lower-cost cathodes to meet future energy storage needs.

## 4 Experimental Methods

### 4.1 Coin Cell Preparation

The laminates of the positive electrode and the Gr negative electrode used in this study were provided by Argonne’s Cell Analysis, Modeling, and Prototyping (CAMP) facility. The details are shown in Table 1.

**Table 1:** Composition of the laminates used in the manuscript. The laminates were coated by an automatic slot-die coater in a dry room and dried under vacuum at 80°C^a^

Positive Electrode	Negative Electrode
(single-sided)	XCEL 2021 Midterm electrode Targeted Round 2 areal capacity. SLC1506T Lot#: 573–824
84 wt % LMR-NM (w/coating)	91.83 Superior Graphite SLC1506T
8 wt % Timcal C-45	2 wt % Timcal C-45 Carbon
4.26 wt % solvay 5130 PVDF Binder	6 wt % Kureha 9300 PVDF Binder
RNGC LMR-NM: 0.3 Li_2_MnO_3_ + 0.7 LiMn_0.5_Ni_0.5_0_2_ (w/wet process coating) electrode, Powder Lot#: AG20220217	0.17 wt % Oxalic Acid
SS-single sided → calendered	SS-single sided → calendered
Total Electrode Thickness 74 μm; SS Coating Thickness: 54 μm	Total Electrode Thickness 80 μm; SS Coating Thickness: 70 μm
Porosity: 41.6%	Porosity: 34.4%
Total SS Coating Loading 11.15 mg/cm^2^	Total SS Coating Loading 9.9 mg/cm^2^
Total SS Coating Density, 2.06 g/cm^3^	Total SS Coating Density, 1.42 g/cm^3^
Estimated C/10 a real capacity 2.19 mAh/cm^2^. [After activation, reversible C/10 of 234 mAh/g for 3.0 to 4.5 V vs Li/Li^+^.]	Estimated C/10 areal capacity 3.16 mAh/cm^2^. [Based on rev. C/10 of ∼330 mAh/g from 0.005 to 1.5 V vs Li/Li^+^.]

a They were dried at 100 °C under vacuum in an Ar-filled glovebox right before use.

The microporous separator Celgard 2325 was used in the coin cell assembly. All electrodes were dried at 110°C under vacuum in an Ar-filled glovebox prior to use, and coin cell parts (except the separators) were dried in an oven at 100°C. The separators were dried at 50°C overnight. The 2032-type coin cells were assembled in an Ar-filled glovebox. The diameters of the positive electrode, graphite electrode, and separator were 14, 15, and 16 mm, respectively. The discs were cut by using a manual cutter. The total amount of electrolyte added was 25 μL for each cell, and the N/P ratio was ≈1.4. In our study, at least three individual cells were tested for each electrolyte. A CR2032 coin cell (www.predmaterials.com) was used, and VWR Signature Ergonomic High Performance Single Channel Variable Volume Pipettors were used together with VWR tips. No extra wetting was required for the assembled cells. The cells were made in triplicate and cycled in an environmental chamber (convection heating) at a constant temperature of 30°C.

### 4.2 Cycling Protocol

The design of the protocol of Gr||LMR-NM is critical for the identification and development of viable additives for improvements. The protocol used comprises the following steps, adapted from previous work with some modifications to reduce the duration of one complete cycle.^30^

Formation step of 3 cycles at C/10 cycles from 2.5 to 4.3 V, followed by activation cycles 2.5–4.6 V. For LMR-NM positive electrodes, using a standard active weight of 14.4 mg, the current used for 1C rate is 2.8 mA, assuming the theoretical capacity for LMR-NM is 194 mAh g^*–*1^. C/10 is 280 μA.
Reference performance test (RPT) of C/25. Please note that the revised protocol incorporates a slower rate compared to our previous results on the LiNi_0.9_Mn_0.05_Co_0.05_O_2_ study.^43^ This slower Rate Performance Test (RPT) is specifically designed to counteract the impedance effect observed at higher C rates, while simultaneously ensuring the generation of distinct and well-defined voltage plateaus. For lithium nickel man- ganese oxide (LNMO) positive electrodes, using a standard active weight of 14.4 mg, the current used for 1C rate is 2.4 mA, assuming the theoretical capacity for LMR-NM is 194 mAh g^*–*1^. C/25 is 112 μA.
Preparation cycle of 1/C and one Hybrid Pulse Power Characterization (HPPC) cycle.^44^ The HPPC cycle is composed of 10 s pulses of 2C discharge and 1.5C charge, each followed by a 40 s rest. The currents in this context are expressed as C-rates, where 1C = approximately 1.7 mA.
Aging cycles at a rate of C/3; the upper cutoff voltage is 4.4 V with no hold and a lower cutoff voltage of 2.5 V. C/3 is 933 μA.
Steps 2–4 were repeated for a total of 4 loops.
The last RPT cycle of C/25 followed by one HPPC cycle.

Note: Cell activation was performed at 4.6 V, as previously demonstrated for active Li_2_MnO_3_ → Li_2_O + MnO_2_ to increase the volume of cyclable lithium, which stabilized the lithiated 0.3Li_2_MnO_3_·0.7LiMn_0.5_Ni_0.5_0_2_ electrodes. The discharge specific capacity, which fully accounts for the formation of 0.3MnO_2_·0.7LiMn_0.5_Ni_0.5_0_2_, is approximately 288 mAh/g.^45^ Since the 4.6 V upper cutoff voltage is only associated with activation of Li_2_MnO_3_, the subsequent cycles were reduced for monitoring activity, similar to the other cobalt-free spinel cathode studies.^46,47^ Each electrolyte formulation was tested with triplicate cells, and the averages of both specific capacity and areal specific impedance were used. The cycling performance with specific capacity is shown in Figure S9.

### 4.3 Cryo-FIB Sample Preparation

Cross-sectioning of cycled cathodes to prepare samples for cryo-STEM was performed at the Center for Integrated Nanotechnologies (CINT) based on methods used in previous work.^34^ CINT offers a Leica cryo-suite including a loading station, cryo-transfer shuttle, and sputter coater, all capable of cryo with inert transfer, along with an LN_2_-cooled SEM stage and cryo lift-out needle inside a Thermo Fisher Scientific Scios 2 FIB/SEM. The cathode was removed from the coin cell, cut with scissors into a small triangle, and mounted on the SEM stub at room temperature inside an argon-filled glovebox using copper tape. The Leica cryo-transfer shuttle was then used to remove the mounted sample from the glovebox, air-free, at room temperature. A 10 nm Pt thin film was sputtered over the entire sample inside the Leica cryo-sputter coater under high vacuum to further reduce charging under the electron and ion beams. Air-free transfer was completed with loading through a port into the Leica cryo-stage of the FIB/SEM.

During FIB/SEM, beam voltages and currents were kept low to reduce damage and heating; the electron beam was kept at or below 5 kV and 50 pA, and the Ga-ion beam at or below 16 kV and a few nanoamps. Final thinning was done at 16 kV and less than 150 pA. Sample attachment to the lift-out needle was completed at room temperature using organometallic Pt patterning with fine ion-beam milling steps to ensure a robust connection, followed by attachment to a copper half-grid on two sides of the lifted-out section of ∼2 μm thick. The sample was then cooled on the stage in the high-vacuum environment of the SEM to −150°C for the completion of the FIB polishing to thin a window in the center of the sample to electron transparency. Beam currents below 150 pA and small tilts of ± 1–2° were used to thin the window to ∼100 nm thickness, followed by a final polish at 8 kV at 75 pA using ± 2° tilts to remove most of the surface FIB damage. Once thinned, the sample was removed from the SEM. The sample and stage were allowed to return to room temperature in the Leica inert transfer shuttle and returned to the Ar-filled glovebox. The sample was sealed in a glass jar under argon until it was ready for STEM imaging.

### 4.4 Cryo-STEM with EELS Data Acquisition

To prepare for cryo-transfer to the STEM, within the Ar-atmosphere glovebox, the sample was sealed tightly in a screw-top cryo grid storage box from Ted Pella, which was then sealed within a glass jar. The jar was removed from the glovebox and opened, and the grid storage box was immediately dumped into a dewar of LN_2_. The grid box was then opened with a screwdriver while submerged under the cryogen. This cryo-transfer method was similar to what has previously been applied in cryo-EM of battery materials;^48^ however, here a cryo grid box was used to seal the sample instead of a plastic tube. The plastic tube method requires breaking the tube with pliers after plunge-freezing, which caused concerns over damaging or snapping off the delicate FIB lamella. Unscrewing the grid box lid was a much gentler process and still doable under LN_2_, and the lamella was kept in good condition.

After freezing, the grid was loaded into a Gatan 626 cryo-transfer holder, which had been precooled to below −170°C, using the Gatan cryo-transfer station. The holder was then loaded into a Cs-corrected Spectra 200 STEM for characterization. HAADF imaging and EELS acquisition were performed at 200 kV with a 24.2 mrad convergence angle. The screen current was maintained at approximately 20–30 pA to limit the electron dose. EELS maps were acquired using a Gatan Model 977 Enfinium ER EELS spectrometer. Maps were acquired with 0.25 eV/ch dispersion and 2 s pixel time in DualEELS mode, with simultaneous acquisitions beginning just before the zero-loss peak and the O K-edge (532 eV).

### 4.5 EELS Data Analysis

Analysis of the EELS data described here was carried out with Python scripts. The energy axis for both the low- and high-loss regions for each pixel (1–2 nm) was calibrated using the zero-loss peak to accurately map and analyze edge fine structures across the field-of-view.

The intensity of the HAADF image was used in an Otsu thresholding method to define the particle surface; then, a distance map was created by calculating the shortest distance from each pixel to the surface.^49^ The surface of the cathode particle was defined as zero, the particle interior was defined in the positive direction, and the CEI/carbon PVDF binder was defined in the negative direction (Figure S2). Pixels were then binned together by distance to increase the signal-to-noise ratio. The background intensity of the manganese (Mn) L_2_,_3_-edges and the oxygen (O) K-edge were subtracted by fitting an inverse power law function to a 30–40 eV window before the respective edge onset and extrapolating the fit function to the edge energy range.^50^ The Mn L_2,3_-edge peaks from the binned data were fit using a least-squares fitting method to two Gaussian functions to extrapolate peak position, intensity, and separation for valence state determination (Figure 2).

The white line intensity ratios of the Mn L_2_/L_3_ edge peaks were calculated for the binned distance data. Then, a calibration curve for mixed-valence Mn minerals from Loomer et al. (*y* = 0.68*x* ^2^ – 5.02*x* + 11.27; *R* ^2^ = 0.997)^35^ was applied to estimate the Mn valence across the cycled LMR-NM cathodes. It is worth noting that multiple calibration curves^35,39,40^ from the literature were used to calculate Mn valence, resulting in similar trends in Mn valence state changes across cathode particles but different absolute values of the valence (Figure S5). The curve from Loomer et al. was chosen for reporting in the main text, as it had the largest and most relevant sample size; a detailed discussion of the impacts of the choice of reference for valence state calculation is provided in the Supporting Information.

The O K-edge was used to define the CEI thickness for each cycled cathode. The O K-edge was plotted as a function of distance for all spectrum images (as shown in Figure 4), and it was qualitatively determined where the edge disappeared. This distance was then averaged over all spectrum images for each sample (4–6 values per sample) to estimate the CEI thickness for that sample. Figure S7 shows the average distance at which the Mn L_2,3_-edge disappeared for all samples, calculated in the same way. While this method is more susceptible to outliers than only including Mn valence data points that are the average of at least three spectrum images, the general trends remain that LiDFOB-containing samples show Mn present to the same distance as that of O, while all other samples show Mn loss near the surface of the CEI.

The average relative thickness of cryo-STEM samples over the region of interest for Mn and O edge analyses was calculated by computing the log-ratio relative thickness at each pixel in digital micrographs for all EELS spectrum images. A line scan was drawn along the surface of the particle with an integration width of ∼20 nm in each direction (into or out of the particle). Values along the line scan were averaged to yield an average relative thickness for the spectrum image. Finally, the individual averages from all spectrum images were averaged for each sample; these are reported in Table S1 and discussed in the Supporting Information.

### 4.6 Inductive Coupled Plasma-Mass Spectroscopy

To study the dissolution of the cathode and the migration of transition metal ions to the anode, the anode material was washed, placed in a quartz beaker, and heated in a furnace at 700 °C for 12 h to eliminate organic materials and carbon. The resulting ash was treated with a reflux of nitric and hydrochloric acids at 220 °C for 1 h, followed by dilution with water. The samples were examined using ICP-MS to measure the concentrations of transition metals, which were then adjusted based on the anode’s weight. The measurements were conducted with a PerkinElmer NexION 2000 ICP Mass Spectrometer, calibrated using standards traceable to the National Institute of Standards and Technology

## Supplementary Material



## Notes

Further details on EELS analysis (including Mn valence calibration), ICP-MS results, and cycling protocol in Figures S1–S9, and Table S1 (PDF)
