# Supplementary material for: Influence of Electrolyte Additives on Interfacial Stability of Manganese-Rich Lithium-Ion Battery Cathodes
Source: ACS Appl Energy Mater. 2025 Aug 6;8(16):11873–83. doi: 10.1021/acsaem.5c00862 (PMC12381821; doi:10.1021/acsaem.5c00862)
Supplement: Supplementary file 1 [file ae5c00862_si_001.pdf]

Supporting Information

Influence of electrolyte additives on interfacial stability of manganese-rich lithium-ion battery cathodes

Nikita S. Dutta,*,†,1 Madison King,†,1,2 Bingning Wang,3 Chen Liao,*,3 John S. Mangum,1 Donal P. Finegan,1 Bertrand J. Tremolet de Villers,1 and Katherine Jungjohann*,1

[1]*Materials, Chemistry, and Computational Sciences, National Renewable Energy Laboratory, Golden, Colorado 80401, USA*

[2]*Center for Materials Interfaces in Research and Applications, Northern Arizona University, Flagstaff, Arizona 86011, USA*

[3]*Chemical Sciences and Engineering Division, Argonne National Laboratory, Lemont, Illinois 60439, USA*

[†]*These authors contributed equally to this work.*

[*]*E-mail: nikita.dutta@nrel.gov; liaoc@anl.gov; katherine.jungjohann@nrel.gov*

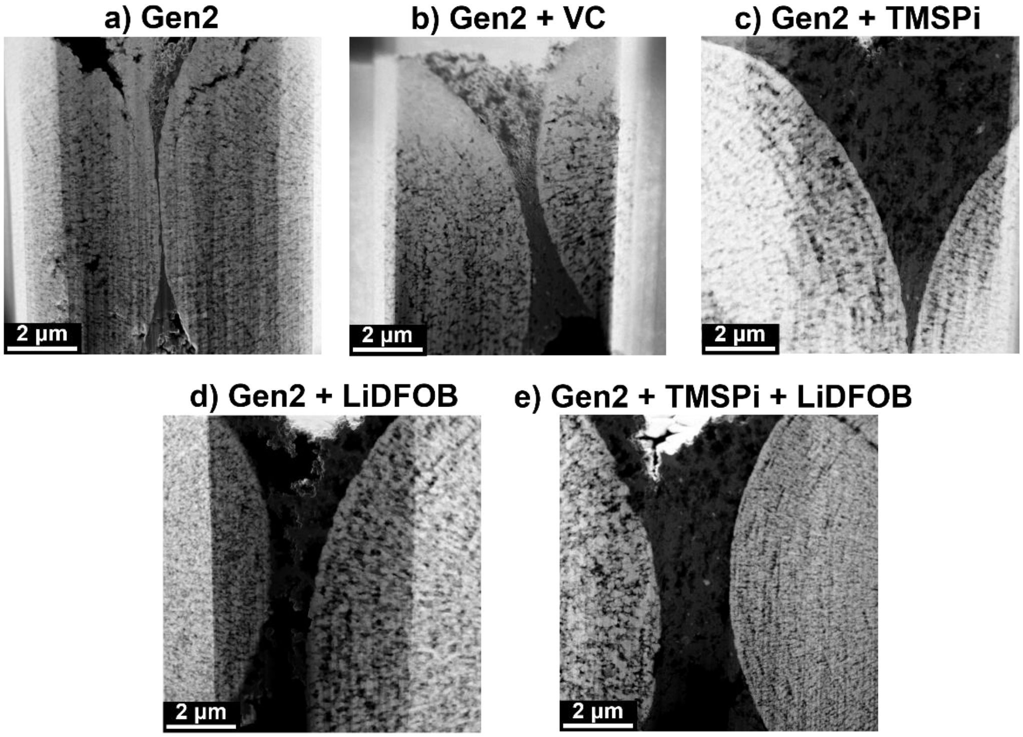

Figure S1. Representative low-magnification HAADF-STEM images of samples cycled in (a) Gen2, (b) Gen2 + 1 wt% VC, (c) Gen2 + 2 wt% TMSPi, (d) Gen2 + 1 wt% LiDFOB, or (e) Gen2 + 2 wt% TMSPi + 1 wt% LiDFOB. Cross-sections captured two individual particles within the cathodes, as well as the carbon PVDF binder in between.

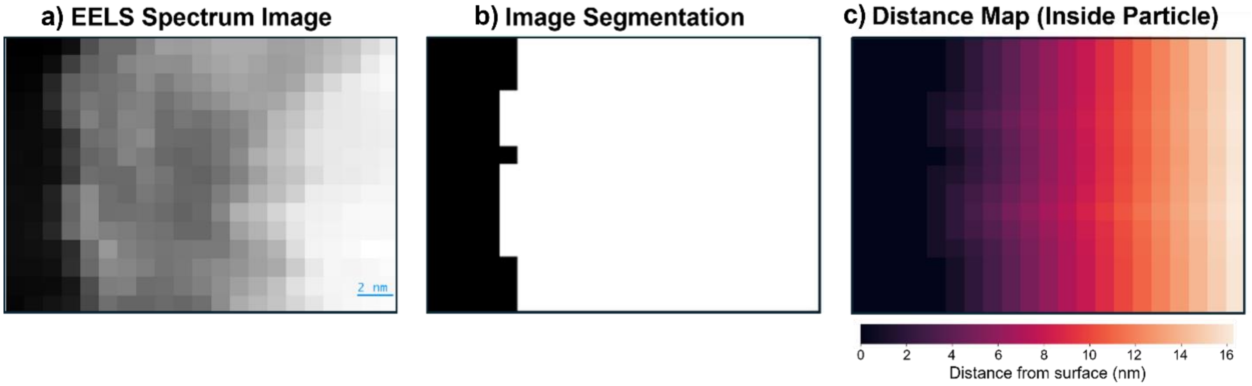

Figure S2. Illustration of image segmentation and distance mapping process used in EELS analysis. a) Original EELS spectrum image contains the HAADF signal, low-loss EELS spectrum, and high-loss EELS spectrum at each pixel. b) An Otsu threshold is applied to the HAADF signal to segment the image and identify the surface of the particle. c) The shortest distance from each pixel to the surface is calculated and used to bin pixels as illustrated by Figure 2 in the main text. The distance map shown here only shows distances inside the particle, defined as positive distances; an equivalent map was prepared for distances outside of the particle, defined as negative distances.

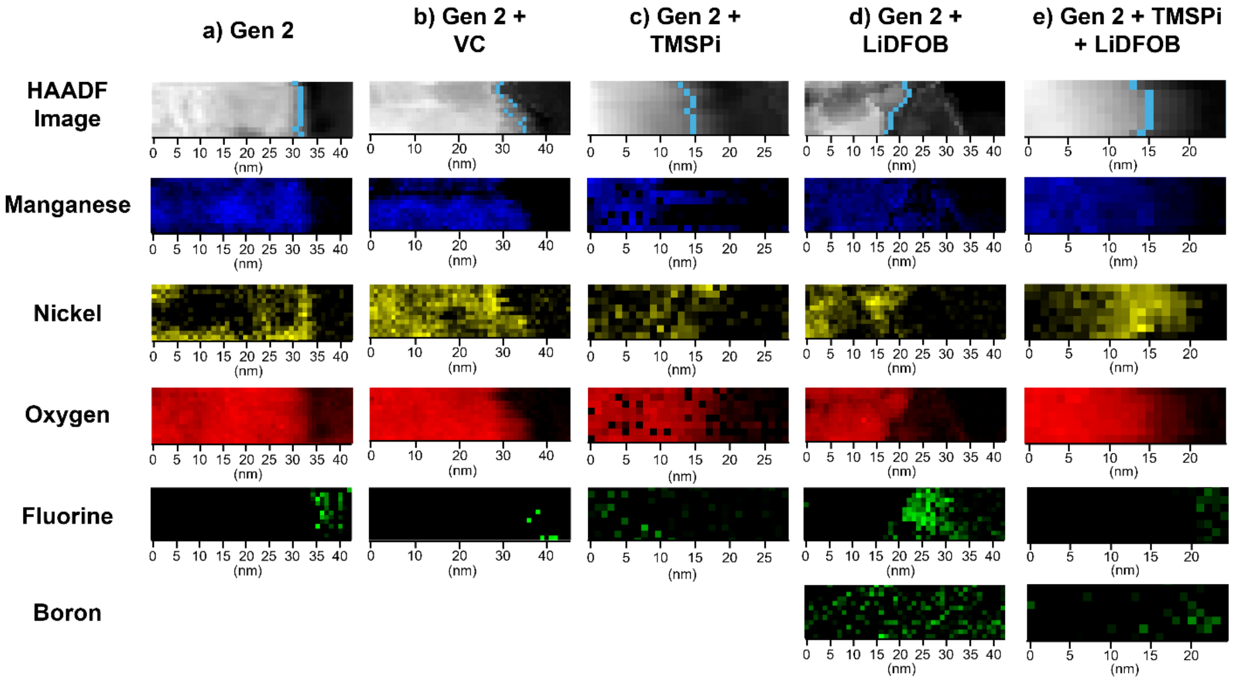

Figure S3. Colored EELS signal elemental maps of (top to bottom) manganese, nickel, oxygen, fluorine, and boron for all cycled cathode samples. The blue line in the HAADF image for each sample represents the particle edge determined by an Otsu threshold. The Mn signal in the (a) Gen 2 and (b) Gen2 + 1 wt% VC is not seen beyond the defined particle surface. Fluorine signal is seen outside of the cathode particle in the CEI and/or carbon PVDF binder execpt for (c) Gen2 + 2 wt% TMSPi. The Mn signal for the samples with (d) Gen2 + 1 wt% LiDFOB and (e) Gen2 + 2 wt% TMSPi + 1 wt% LiDFOB elctrolytes are seen to extend past the defined particle surface into the CEI in agreement with Mn valence vs. distance plots. It is worth noting that boron is distributed differently in the (d) Gen2 + 1 wt% LiDFOB and (e) Gen2 + 2 wt% TMSPi + 1 wt% LiDFOB samples. The uptake of boron into the cathode particle in (d) Gen2 + 1 wt% LiDFOB could be evidence of a non-passivating CEI, contributing to poorer electrochemical performance in comparison to (e) Gen2 + 2 wt% TMSPi + 1 wt% LiDFOB.

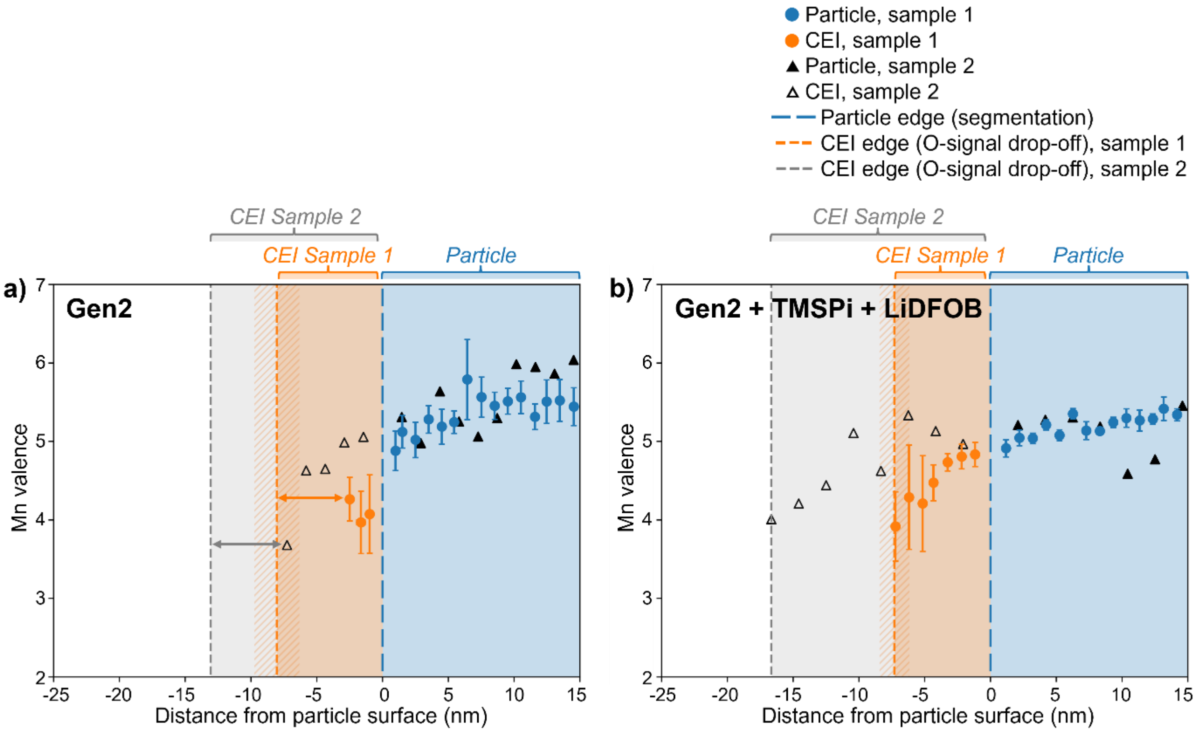

Figure S4. Mn valence plots for the (a) Gen2 baseline and (b) Gen2 + 2 wt% TMSPi + 1 wt% LiDFOB samples, including additional data from a second sample taken from a different cryo-FIB lift-out from a different part of each cathode (black triangles). There is heterogeneity in CEI thickness between samples, with the second sample showing a thicker CEI in both cases. However, the calculated Mn valence is quite consistent between samples of each type, as are the trends related to Mn retention at the CEI surface. In both Gen2 samples, Mn is absent until about ~5 nm within the CEI surface (marked by the gray and orange arrows), while Mn is present throughout the CEI in both Gen2 + 2 wt% TMSPi + 1 wt% LiDFOB samples.

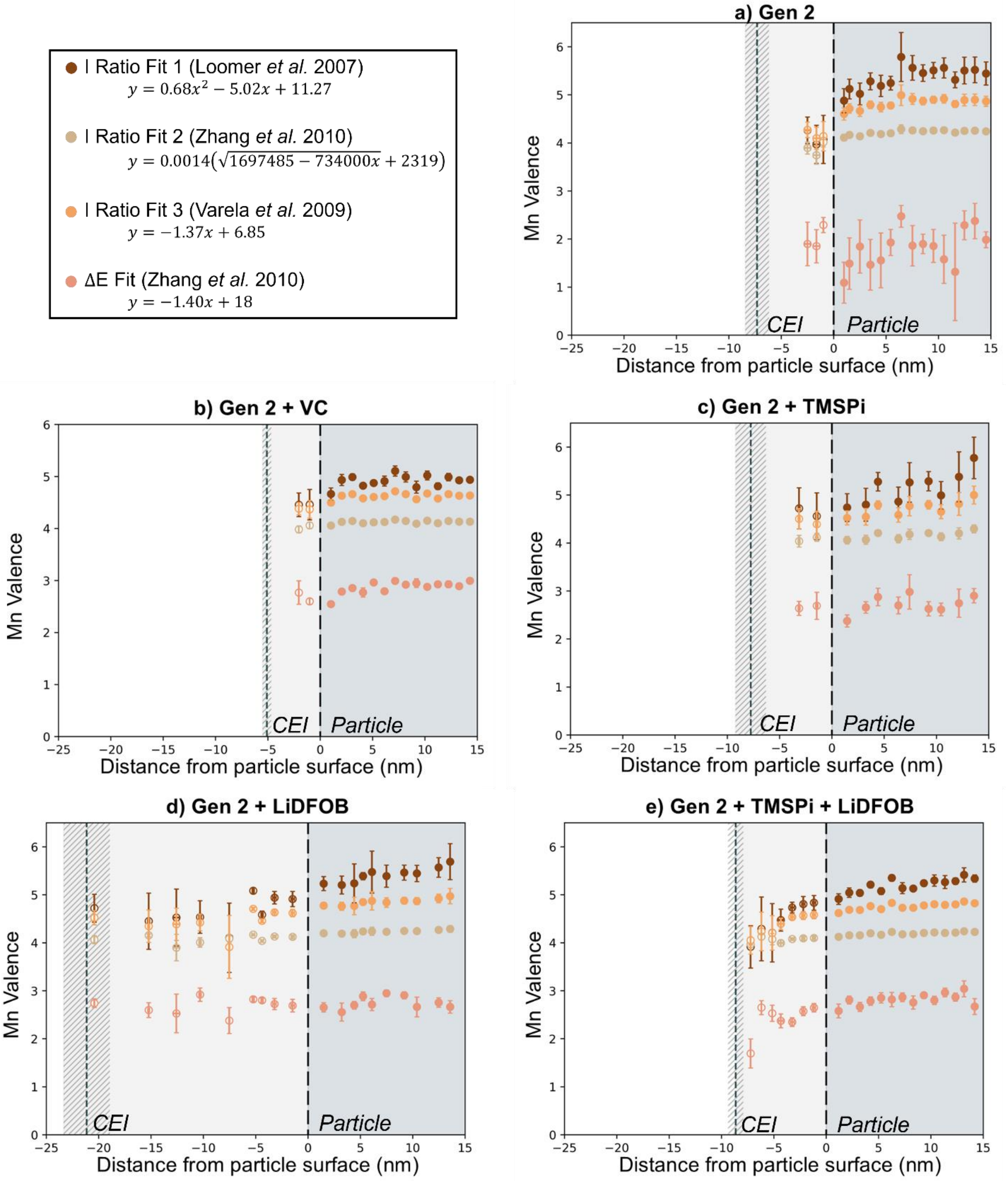

Figure S5. Manganese valence states from the CEI layer to the particle interior determined by different standard fit curves from the literature for all samples: (a) Gen2, (b) Gen2 + 1 wt% VC, (c) Gen2 + 2 wt% TMSPi, (d) Gen2 + 1 wt% LiDFOB, or (e) Gen2 + 2 wt% TMSPi + 1 wt% LiDFOB. The thick dashed line at 0 nm indicates the defined particle surface. Positive distance values (dark grey shaded area) are defined as the particle interior and negative distance values (light grey shaded area) are defined as the particle exterior, including the CEI. The thin dashed line indicates where the O K-edge signal becomes undetectable in the EELS spectra, defining the CEI thickness.

Mn Valence State Calibration Curves

A calibration curve can be used to determine the Mn valence state from the white line intensity ratio of the L_2 and L_3 Mn edges. Fits for these curves can vary due to instrumental and environmental factors. The Mn valence was determined based on calibration curves from the literature, therefore conclusions on the absolute value of the valence states cannot be confidently made. The focus of this work, rather, is to illustrate the variation of Mn valence across the sample, which can be understood from these standards available in the literature. The standard from Loomer et al. was used for reporting in the main text because the mixed metallic Mn oxides used in that work most closely resemble the cathode particles studied here, and because their calibration curve contained more datapoints than other literature, which gave increased confidence in the fit. The work from Loomer et al. also demonstrates that while doing a thickness correction for plural scattering events in samples improves the standard curve fit, it does not significantly change the results. Due to variation of thickness across the samples studied here, the standard curve without correction for plural scattering was used in the main body of this article.

While there is a difference in the absolute value of the calculated Mn valence based on the literature standard used, as shown in Figure S5, the downward trend in Mn valence moving from the particle interior to the CEI is similar regardless of the standard fit applied. It is worth noting that Zhang et al. reports calibration curves for determining Mn valence based on either the white line intensity ratio of the L_2 and L_3 peaks or the energy difference between the peaks, ΔE (L_2-L_3); both methods are shown in Figure S5. While using the ΔE (L_2-L_3) to determine the valence of metal oxides is valid, it is not as accurate due to large errors introduced by broadening of the L_2-edge because of Croster-Kronig Auger decay effects; thus, the intensity ratio is used in the main text.

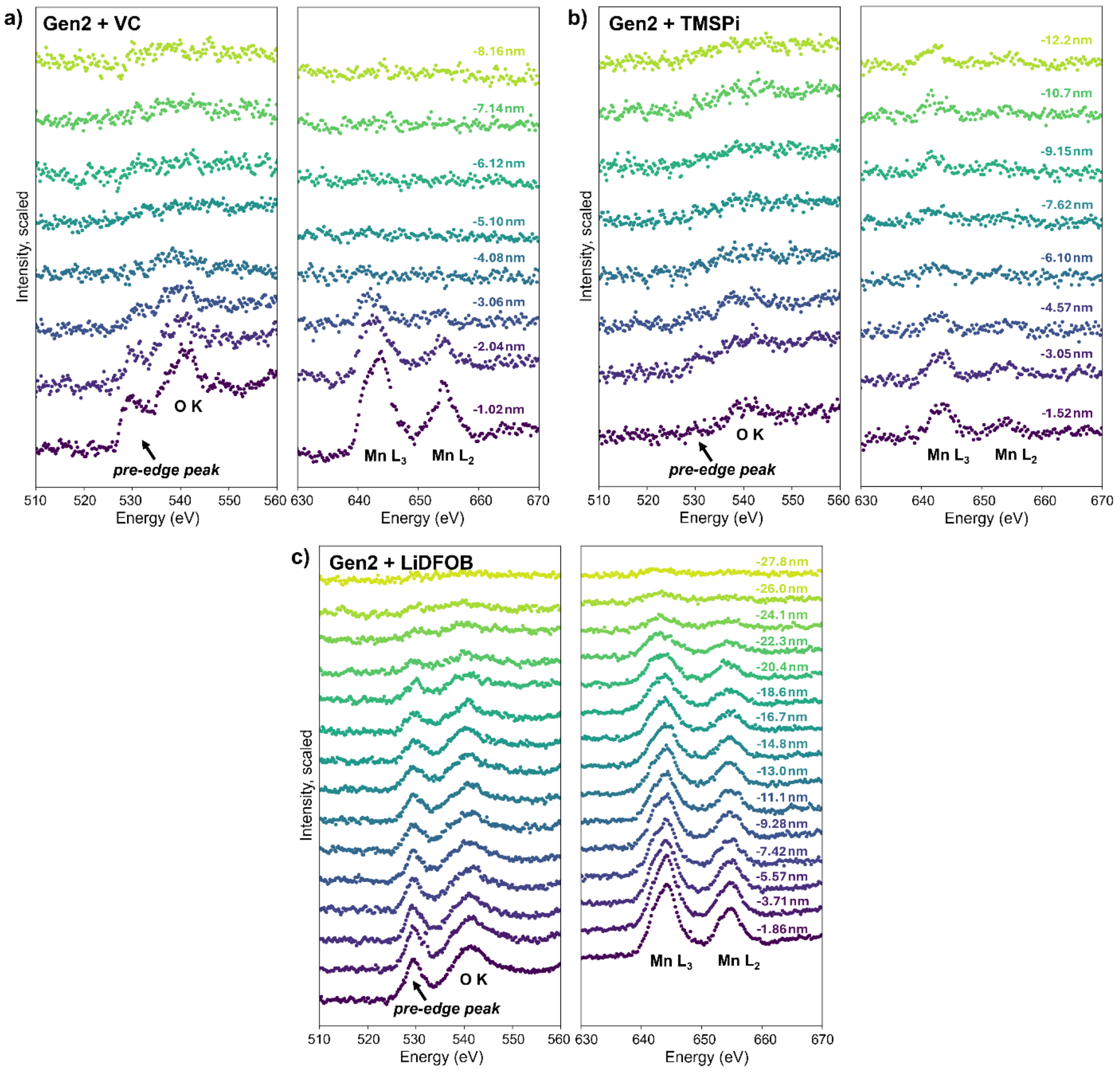

Figure S6. O K-edge (left) and Mn $L_{2,3}$-edge (right) with increasing distance from the particle surface into the CEI for representative regions of samples cycled in (a) Gen2 + 1 wt% VC, (b) Gen2 + 2 wt% TMSPi, or (c) Gen2 + 1 wt% LiDFOB. The pre-edge peak before the O K-edge (around 535 eV) and Mn $L_{2,3}$-edges are most prominent and present furthest from the surface in the sample cycled with LiDFOB, in line with improved Mn retention throughout the CEI. Minor resurgence of the Mn L_3-edge around 10 nm from the surface in the sample cycled with TMSPi (b), after it initially disappears around 6 nm, may indicate a small amount of redeposition of solvated Mn during sample drying.

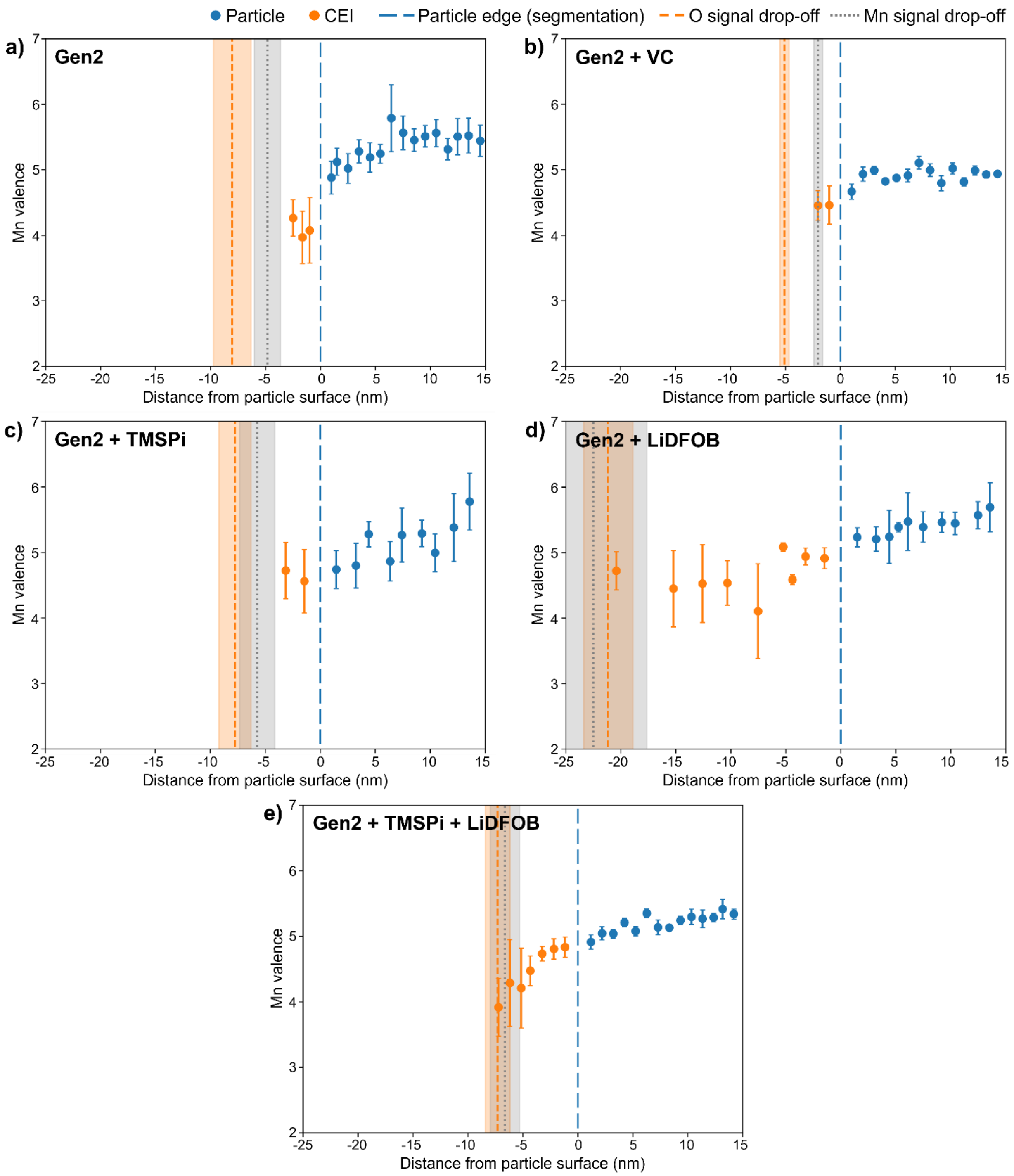

Figure S7. Mn valence state plots with both the O K-edge signal drop-off and Mn $L_{2,3}$-edge signal drop-off plotted for all samples. The (a) Gen2 baseline and (c) Gen2 + 2 wt% TMSPi samples show some discrepancy between the last Mn valence data point (orange circles) and the average point of Mn $L_{2,3}$-edge drop-off (gray dotted line). This is because the average point at which the Mn $L_{2,3}$-edge signal drops off is influenced more strongly by outliers than are the data points, which are only plotted if they are the average of three different regions in the sample (using 1 nm distance bins for averaging). For instance, if only one region in a sample showed

notable Mn signal at a distance greater than -4 nm from the surface, that data influences the average Mn signal drop-off but is not included in the Mn valence data points. This explanation is consistent with the fact that the last Mn valence data point coincides exactly with the average Mn signal drop-off in the Gen2 + 1 wt% VC sample (b), which has the smallest error bars indicating the least heterogeneity between regions. Notably, regardless of whether the last Mn valence data point or the Mn signal drop-off is used to determine if Mn is retained as far into the CEI as O is, only the LiDFOB-containing samples (d, e) show Mn presence fully coincident with O presence, while all other samples show the absence of Mn close to the CEI surface.

Table S1: Average relative thickness near particle surface of EELS maps.

Sample	Average Relative Thickness (mfp)	Standard Error
Gen2	2.0	0.18
Gen2 + 1 wt% VC	1.2	0.15
Gen2 + 2 wt% TMSPi	1.4	0.28
Gen2 + 1 wt% LiDFOB	2.1	0.19
Gen2 + 2 wt% TMSPi + 1 wt% LiDFOB	1.5	0.10

EELS Sample Thickness

The average relative thickness near the particle surface of EELS maps used to prepare Mn valence state plots is given in Table S1. In general, samples are thicker within the particle than is ideal for quantitative EELS; this is a consequence of needing to preserve the CEI and carbon PVDF binder, which thin significantly more quickly during cryo-FIB than the cathode particle itself. However, the sample thickness and thickness variation show no clear trends with the CEI characteristics or Mn retention. Thus, this supports that the major conclusions of the EELS analysis conducted here are not artifacts of sample thickness.

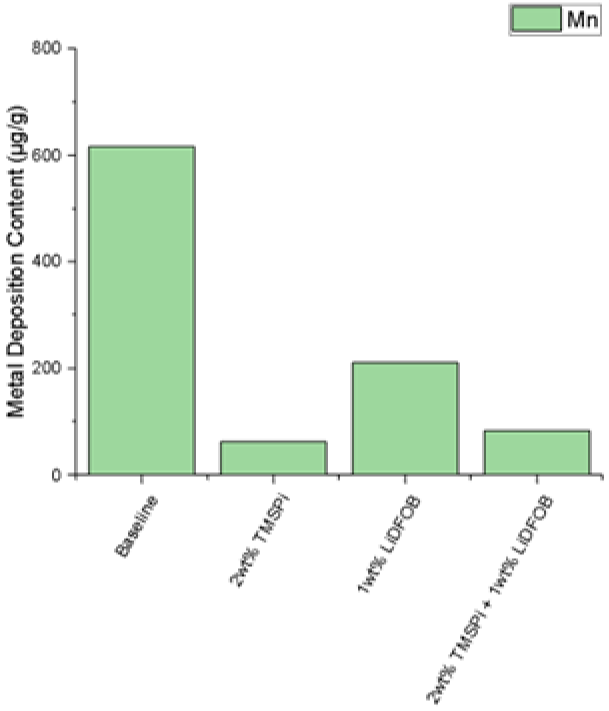

Figure S8. Elemental analysis of cycled graphite anode through inductive coupled plasma mass spectrometry (ICP-MS). Due to the poor performance of VC and high cost, the sample containing 1 wt% VC was not submitted for ICP-MS.

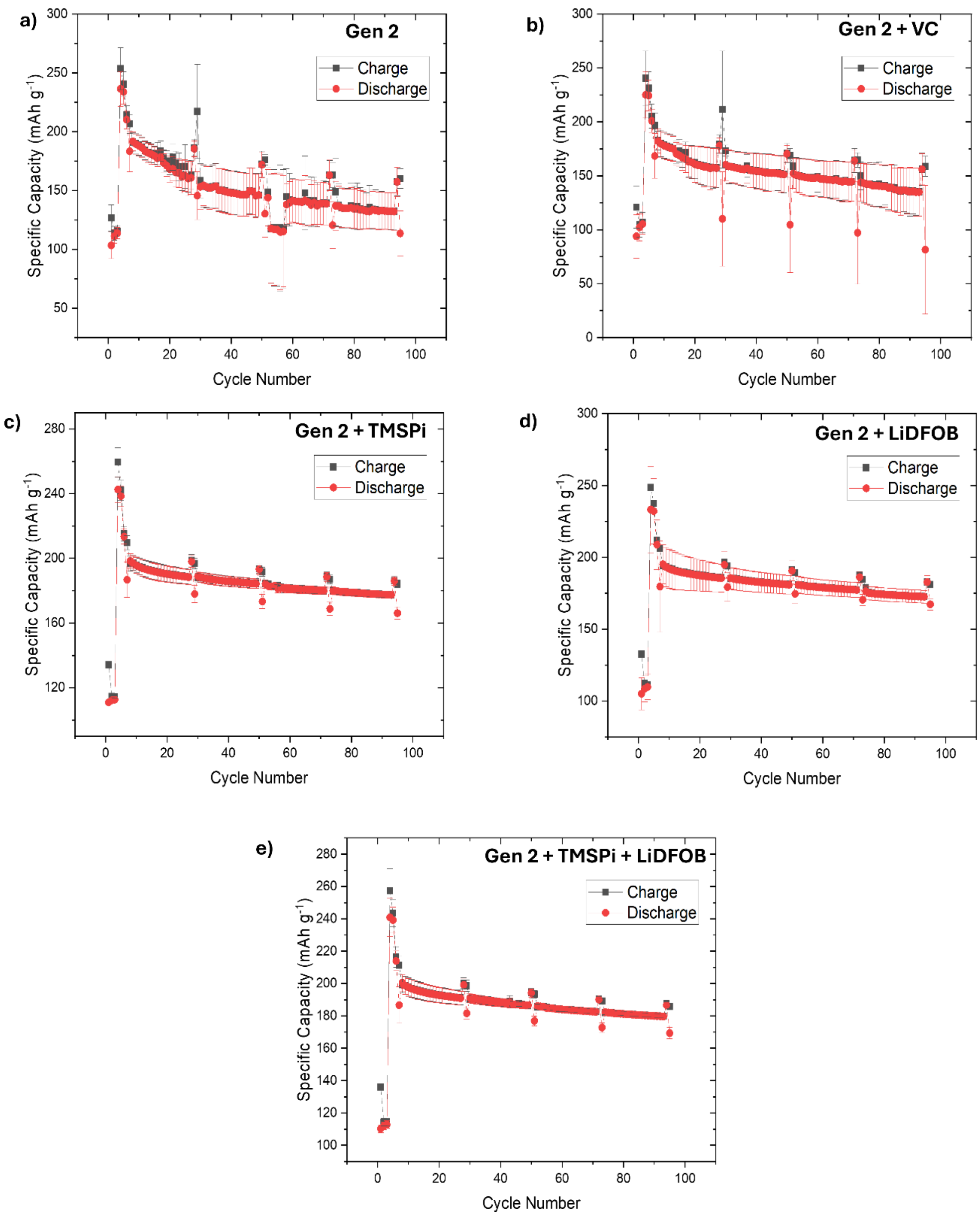

Figure S9. Mean specific capacity vs. cycle number of cells from the charge (black) and discharge (red). Three cells were investigated for each electrolyte additive formulation: (a) Gen 2 baseline, (b) Gen2 + 1 wt% VC, (c) Gen2 + 2 wt% TMSPi, (d) Gen2 + 1 wt% LiDFOB, or (e) Gen2 + 2 wt% TMSPi + 1 wt% LiDFOB. The error bars represent the standard deviation.

References

1. Loomer, D. B., Al, T. A., Weaver, L., Cogswell, S. Manganese valence imaging in Mn minerals at the nanoscale using STEM-EELS. *American Mineralogist*, **2007**, *92*, 72–79.
2. Zhang, S., Livi, K. J., Gaillot, A.-C., Stone, A. T., Veblen, D. R. Determination of manganese valence states in (Mn3+, Mn4+) minerals by electron energy-loss spectroscopy. *American Mineralogist*, **2010**, *95*, 1741–1746.
3. Varela, M., Oxley, M., Luo, W., Tao, J., Watanabe, M., Lupini, A. R., Pantelides, S., Pennycook, S. Atomic-resolution imaging of oxidation states in manganites. *Physical Review B—Condensed Matter and Materials Physics*, **2009**, *79*, 085117.
